# Colitis-associated carcinogenesis: crosstalk between tumors, immune cells and gut microbiota

**DOI:** 10.1186/s13578-023-01139-8

**Published:** 2023-10-24

**Authors:** Junshu Li, Yanhong Ji, Na Chen, Lei Dai, Hongxin Deng

**Affiliations:** Department of Biotherapy, Cancer Center and State Key Laboratory of Biotherapy, West China Hospital, Sichuan University, Ke Yuan Road 4, No. 1 Gao Peng Street, Chengdu, 610041 China

**Keywords:** Colitis-associated carcinogenesis, IBD therapy, Intestinal epithelial cells, Intestinal mesenchymal cells, Immune cells, Gut microbiota

## Abstract

Colorectal cancer (CRC) is the third most common cancer worldwide. One of the main causes of colorectal cancer is inflammatory bowel disease (IBD), which includes ulcerative colitis (UC) and Crohn’s disease (CD). Intestinal epithelial cells (IECs), intestinal mesenchymal cells (IMCs), immune cells, and gut microbiota construct the main body of the colon and maintain colon homeostasis. In the development of colitis and colitis-associated carcinogenesis, the damage, disorder or excessive recruitment of different cells such as IECs, IMCs, immune cells and intestinal microbiota play different roles during these processes. This review aims to discuss the various roles of different cells and the crosstalk of these cells in transforming intestinal inflammation to cancer, which provides new therapeutic methods for chemotherapy, targeted therapy, immunotherapy and microbial therapy.

## Introduction

Inflammatory bowel disease (IBD), which includes ulcerative colitis (UC) and Crohn’s disease (CD), causes long-term immune-mediated colitis-associated colorectal cancer (CAC) [1]. Patients with IBD have an increased risk of developing colorectal cancer (CRC), the third most common cancer worldwide [2, 3]. An extended meta-analysis demonstrated that the risk of CRC is approximately 2% after 10 years and up to 18% at 30 years in UC patients [4]. Therefore, chronic inflammation in IBD patients leads to a significant increase in CRC risk.

Currently, our understanding of the mechanisms leading to a high risk of intestinal cancer in IBD patients has improved [5]. In patients with IBD, the pathogenesis of CRC involves both genes and environmental factors, such as genetic mutations, epigenetic changes and alterations in immune response factors [6–9]. The molecular alterations in colorectal cancer with IBD varied significantly from those in sporadic CRC [10, 11]. First, the timing of gene alterations in colitis-associated colorectal cancer is different from that in sporadic CRC [12, 13]. The APC gene is usually lost at a later stage in CAC, whereas it always occurs at an earlier date in sporadic colorectal cancer [14]. And p53 gene mutation is an early event in CAC, whereas it appears to be a late event in sporadic disease [15]. Second, there are some differences in the frequency of gene mutations between CAC and CRC. Compared with sporadic mutations, APC and KRAS gene mutations are at a relatively low level in colitis-related cancer [16]. This evidence indicates that gene expression and pathway alterations are closely related to CAC progression in specific cells.

Several types of cells, including intestinal epithelial cells (IECs), intestinal mesenchymal cells (IMCs), immune cells and gut microbiota construct the main body of the colon. A few genes with barrier functions in the intestinal epithelium play a significant role in protecting the gastrointestinal tract from pathogen invasion [17–19]. In colitis and colitis-associated carcinogenesis, these cells are disrupted, resulting in damaged IECs, disorganized IMCs and excessive recruitment of immune cells [20, 21]. Patients with IBD and colon cancer have been associated with aberrant function of the epithelial barrier [22, 23]. In addition, IMCs can regulate the development of colon tumors, including intestinal inflammation regulation, epithelial proliferation, stem cell maintenance, angiogenesis, extracellular matrix remodeling and immune responses [24, 25]. Different immune cells such as neutrophils, macrophages and dendritic cells, are activated by chronic accumulation and are recognized as major contributors to gene alterations [26]. Furthermore, the gut microbiota plays a vital role in the modulation of the immune system in chronic inflammatory diseases of the intestine [27, 28]. Therefore, intestinal epithelial cells, mesenchymal cells, immune cells, and gut microflora play a pivotal role in colitis and CAC. In this review, we discuss the various roles of different cells in the transformation of intestinal inflammation to cancer and provide new therapeutic ideas for IBD and colitis-associated colorectal cancer.

## IECs and colitis-associated colorectal cancer

**Fig. 1:**
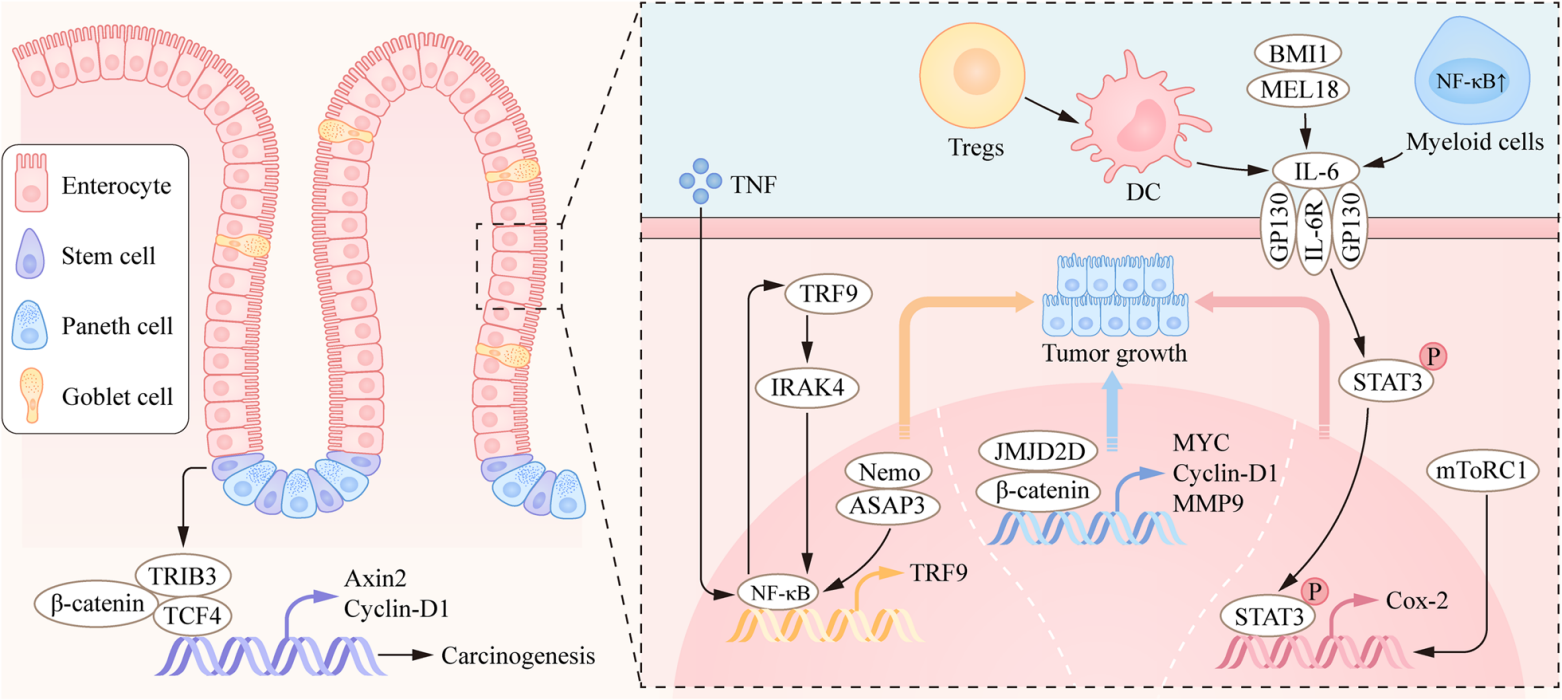
IECs are involved in CAC progression. Many signaling pathways are involved in the occurrence and development of colorectal cancer, such as the NF-kB, Wnt, STAT3 and TGF-β pathways. A few genes, such as ASAP3, promote tumor growth by binding to *Nemo*, while others, such as TRF9, regulate the binding of NF-kB to the promoter region by affecting target genes. Immune cells such as myeloid cells, Tregs, and DCs can regulate the phosphorylation of STAT3 to affect tumor growth. In addition, the Wnt signaling pathway also affects tumor progression. JMJD2D protein can regulate the transcription of Wnt-related genes by binding to β-catenin. Similarly, TRIB3 can also regulate the occurrence and development of tumors in intestinal stem cells by affecting Wnt-related genes

The epithelium is a single-cell layer consisting of various subtypes of particular IECs, such as cup cells, tuft cells, absorptive cells, enteroendocrine cells, M cells, Paneth cells and goblet cells [29]. These cells have pivotal and distinctive functions in maintaining intestinal homeostasis [30]. Paneth cells stay in the small intestine with the function of secreting antimicrobial peptides and maintaining the niche of stem cells in the intestine [31, 32]. In contrast, goblet cells are stayed in the large intestines and produce abundant glycosylated proteins, such as Muc2 [33]. Intestinal epithelium cells (IECs) preferentially absorb nutrients and have a protective barrier effect and strong defense ability against a harmful intestinal microenvironment [34, 35]. Disruption of the intestinal epithelium is a hallmark of IBD. Moreover, the process of gene alterations and pathway changes in intestinal epithelial cells has been demonstrated during the formation of IBD [36–39]. Intestinal tumors originate from gut epithelial cells and develop from gene mutations in a few signaling pathways, such as NF-κB, Wnt, STAT, endoplasmic reticulum (ER) stress and transforming growth factor (TGF)-β (Fig. 1) [40–42].

The NF-κB pathway in the intestinal epithelium is the first pathway in our discussion. Colonic organoid experiments can assess the epithelial responses to inflammatory cytokines in an in vitro model. In colonic organoids, NF-κB signaling is activated in patients with chronic inflammation, which leads to the occurrence of colitis-associated colorectal cancer [43]. In CD patients, NOD2 is a dominant genetic risk factor, accompanied by many rare variants and three main risk-conferring variants [44, 45]. NOD2 regulates TLR signaling and NF-κB pathways, which increases the risk of IBD [35]. Recent studies have suggested that P65 (an important component of NF-κB) binds to the N-terminus of ITF2 to inhibit ubiquitination, promotes the stability of ITF2 and reduces colitis-associated colorectal cancer[46]. Moreover, NFKBIZ (also known as IκBζ) gene mutations were abundant in colitis samples, but the incidence and severity of colorectal tumors decreased significantly in NFKBIZ-deficient mice [47]. The gene deficiency of *Nemo* (NF-κB essential modifier) in the epithelium causes epithelial cell apoptosis and affects the expression of antimicrobial peptides, leading to bacterial migration to the mucosa. In addition, ASAP3 interacts with *Nemo* to regulate the expression of NF-κB, which is associated with poor prognosis and plays an oncogenic role in colorectal carcinogenesis [48]. Moreover, chemotherapy can activate NF-κB and IRAK4 by increasing the transcription of TLR9. Meanwhile, the expression of TLR9 is also inhibited by IRAK4 or IKK inhibitors, which can protect colorectal cancer cells from chemotherapy drugs through a feedforward pathway [49].

The activation of the Janus kinase (JAK)/signal transducer and activation of the transcription (STAT) pathway is important in IEC disorders [50, 51]. STAT3 plays a crucial role in maintaining intestinal homeostasis and strongly protects against chemically induced colitis [52]. Mice with reduced STAT3 activity were highly susceptible to colitis by regulating the IL-6ST/gp130 cytokine receptor, which plays a key role in promoting intestinal barrier function and epithelial regeneration [53, 54]. Conditional knockout mice with a specific STAT3 or ATG16L1 deficiency in IECs can affect the secretion of IL-22, which is associated with wound healing and has a high risk of developing colitis [55–57]. However, abnormal STAT3 activation is also closely related to the malignant progression and pathogenesis of solid tumors such as CAC [58]. The activation and translocation of STAT3 into the nucleus promote the transcription of target genes related to cell proliferation, metastasis and inflammatory response. In colon epithelial cells, BMI1 and MEL18 can promote proliferation and reduce apoptosis to accelerate the development of CAC by regulating the secretion of IL-6/11, which is a regulator of STAT3 [59]. During CAC development, overexpression of the CAMK2γ gene facilitates the activation of STAT3 in the epithelium, thereby promoting the survival and proliferation of IECs [60]. Additionally, the oncogene RXRα is an effective regulator of the inflammatory response that promotes colorectal tumorigenesis by activating the NF-κB-IL-6-STAT3 signaling cascade [61]. Moreover, mTORC1 can activate COX-2 transcription by phosphorylating STAT3 and enhancing the interaction with COX-2 promoter in the colonic epithelium, thus recruiting T helper-17 (Th17) cells and promoting tumor growth [62]. Blockade of IL-6 levels in IECs can inhibit the activation of STAT3 and abnormal cell proliferation, which provides new therapeutic potential [63, 64]. Thus, these studies indicate that STAT3 plays a protective role by maintaining epithelial cell proliferation during acute colitis, while abnormally activated STAT3 promotes the progression of CAC.

The third pathway in our discussion is the Wnt/β-Catenin signaling pathway, which is essential for the pathogenesis of CAC [65]. Whole-exome sequencing analyses have indicated that Wnt pathways play a predominant role in IBD-associated colon tumorigenesis [66]. There is an important correlation between the activation of Wnt and the expression levels of a few genes in intestinal epithelial homeostasis [67–69]. In the CAC mouse model, MUC1-C can form a transcription complex with MYC and act on the LGR5 promoter region, thereby activating LGR5 expression and tumor growth [70, 71]. In addition, histone demethylase JMJD2D is highly expressed in tumors, regulates a few signaling pathways (including Wnt/β-Catenin and Hedgehog) and activates the transcription of downstream target genes related to proliferation, migration, and invasion, which leads to the formation of CAC [72, 73]. In colorectal cancer stem cells, TRIB3 can interact with β-catenin and Tcf4 to increase AOM/DSS-induced colorectal tumor formation and xenograft tumor growth in mice [74]. Currently, researchers are exploring drugs that can inhibit key signaling pathways. For example, Zeng et al. found that scutellarin improved colitis-related colorectal cancer by reducing Wnt/β-Catenin signaling [75]. Researchers also found that synbiotics can significantly inhibit abnormal activation of the Wnt signaling pathway in an AOM/DSS-induced mouse model and alleviate the progression of CAC [76].

Changes in gene expression may affect the development of inflammatory bowel disease [77–79]. The MEP1A gene, which encodes the α subunit of meprins, has a strong relationship with UC patients. In IECs, the subunit of meprins is associated with the transmembrane β subunit and cleaves different substrates, which suppresses the development of UC. Mep1A-deficient mouse models are more susceptible to chemically induced colitis, in accordance with the decreased expression of MEP1A in UC patients [80]. In addition, it has been reported that hepatocyte nuclear factor 4α (HNF4α), a nuclear transcription factor encoded by the gene HNF4α, is crucial for epithelial tight junctions and intestinal permeability by regulating several cytokines and signaling pathways. In IBD patients, HNF4α has low expression, which is consistent with the results in mice lacking HNF4α in IECs, indicating that IECs are more susceptible to drug mediated colitis in mouse models [81]. Moreover, FAM3D (a cytokine-like molecule) is highly expressed in the gastrointestinal tissues and is associated with colonic mucosal integrity, epithelial cell proliferation, antibacterial effects and the development of inflammatory bowel disease [82]. A few genes, such as BRG1 and SETD2, attenuate inflammation in CRC by modulating oxidative stress [83, 84]. E-cadherin, a cell adhesion molecule expressed in epithelial cells encoded by the gene CDH1, plays an important role in cell growth, proliferation, and epithelial differentiation. In addition, a typical pathological characteristic of IBD patients is the loss or disorder of E-cadherin accompanied by increased epithelial permeability [85]. These differentially expressed genes and signaling pathways have the potential to become targets for the diagnosis and treatment of IBD.

## IMCs in CAC progression

**Fig. 2:**
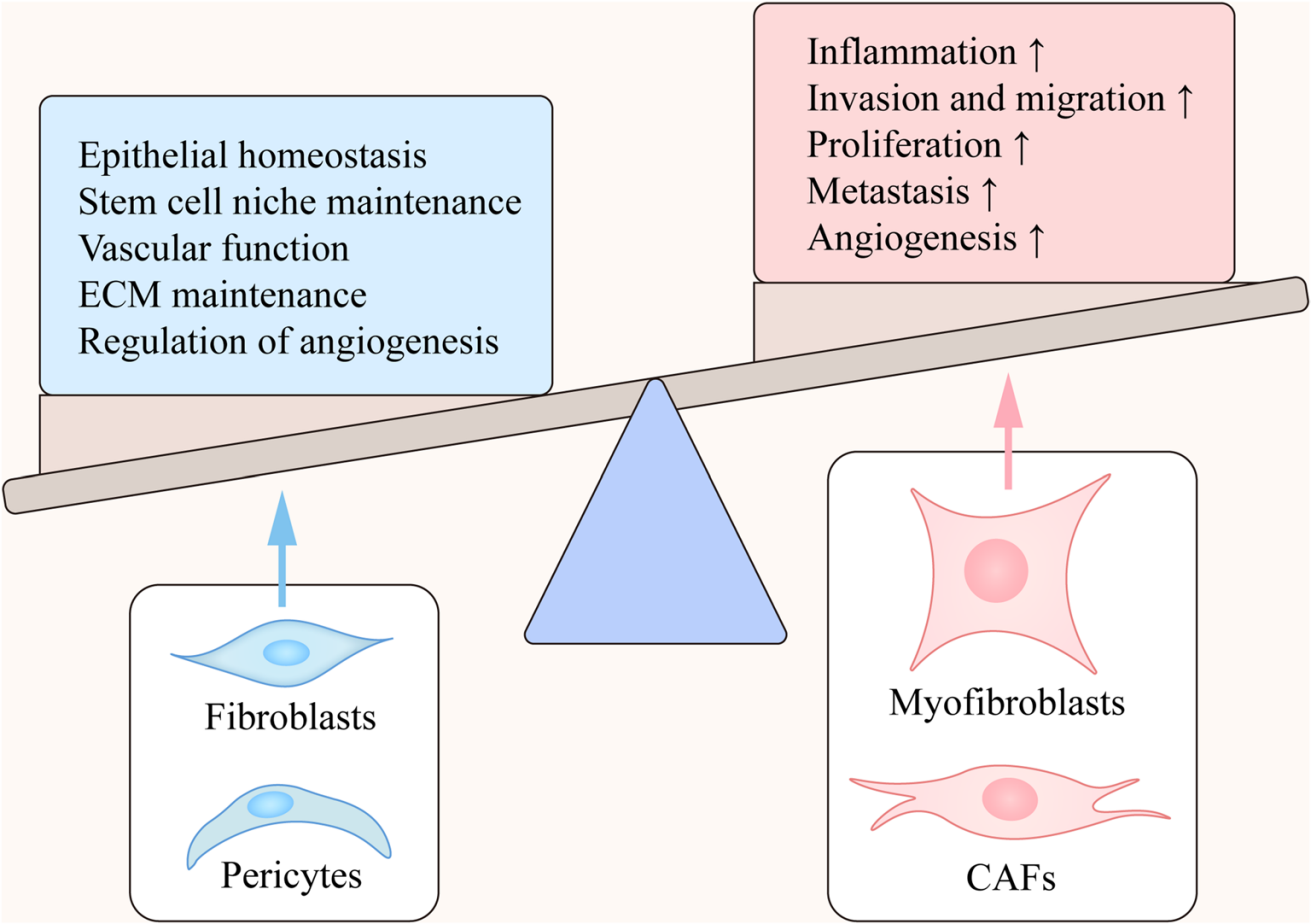
The main function of IMCs in intestinal homeostasis. Intestinal mesenchymal cells play an important role in maintaining intestinal homeostasis. The main functions of fibroblasts and pericytes are maintaining epithelial homeostasis, stem cell niche, vascular function and ECM. Meanwhile, CAFs and myofibroblasts can promote inflammation, cell proliferation, angiogenesis, invasion and migration

Intestinal mesenchymal cells (IMCs) are major components of the normal intestinal tract and intestinal tumors [86]. They include numerous cell types with a similar origin, function and molecular markers, such as intestinal fibroblasts, myofibroblasts and pericytes [87] (Fig. 2). Recent studies used unbiased single-cell profiling involving over 16,500 colonic mesenchymal cells and revealed four subsets of fibroblasts, including TNF superfamily member 14 (TNFSF14), fibroblastic reticular cell-associated genes, IL-33, and lysyl oxidases, which express different transcriptional regulators and signaling pathways [88, 89]. Activated IMCs can promote inflammation and tumor progression by directly affecting the growth of neoplasms and changing the microenvironment of the surrounding tumors. Cancer-associated fibroblasts (CAFs) consist of a population of cells from different origins that lead to tumor initiation, progression, metastasis and poor outcomes of patients via interactions and changes in the microenvironment [90, 91].

### Fibroblasts

Fibroblasts reside in the lamina propria with α-smooth muscle actin (α-SMA)-negative characteristics, are adjacent to the intestinal epithelium and remain in the quiescent phase with poor transcriptomic and metabolic activities in the normal colon [92]. Fibroblasts have different characteristics than epithelial and immune cells and possibly originate from a mesenchymal lineage. They are connective tissues that can synthesize collagen components [93]. The main functions of fibroblasts include the accumulation and maintenance of the extracellular matrix (ECM), stabilization of adjacent epithelia and regulation of inflammation [92, 94]. Many studies have shown that fibroblasts can be activated by multiple factors, such as growth factors, inflammatory cytokines and chemokines, mechanical stress and reactive oxygen species in colitis and colorectal cancer [95, 96]. The secretion of periostin in fibroblasts can promote the occurrence and development of colorectal cancer by activating FAK-Src kinase, the Yap/TAZ pathway and IL-6 expression in tumor cells [97]. In addition, IL-11^+^ fibroblasts can activate tumor cells and fibroblasts by producing a large amount of IL-11 and promote tumorigenesis at the same time [98]. Furthermore, fibroblasts in tumors include activated fibroblasts (myofibroblasts), cancer-associated fibroblasts (CAFs) and cancer-associated mesenchymal stem cells (MSCs) [99].

### Myofibroblasts

Myofibroblasts are stellate shaped, proliferate and are usually more active, which makes them different from quiescent fibroblasts in terms of morphology and metabolism [100]. The typical characteristics of intestinal myofibroblasts are the expression of α-SMA, CD90, and vimentin. Among these markers, α-SMA is considered the most typical intestinal myofibroblast marker. However, α-SMA is also expressed in pericytes, partial smooth muscle cells and bone marrow-derived mesenchymal stromal cells [101]. Myofibroblasts can perform various functions by regulating signaling pathways and cytokines. MyD88 signaling in myofibroblasts promotes CAC progression by activating osteopontin secretion, macrophage M2 polarization and the STAT3/PPARγ pathway [102]. Activated fibroblasts can produce MMPs, which are ECM-degrading proteases and promote cancer cell invasion. For example, the overexpression of MMP1 induces invasiveness, and MMP3 expression promotes epithelial-to-mesenchymal transition (EMT) progression and the invasion of cancer cells into adjacent tissues [103]. Studies conducted in Tnf^ΔARE/+^ mice have shown an increase in the expression of MMP9 and ICAM1, thereby inducing ECM remodeling and adaptive immune responses [104, 105]. Activated fibroblasts play a role in regulating immune homeostasis, including immune cell recruitment and modulation by secreting chemokines and infection- or injury-related cytokines. Additionally, myofibroblasts can maintain epithelial homeostasis by sensing the inflammatory environment created by the tissue or bacteria and mediate epithelial regeneration through the activation of the Cox-2 signaling pathway [106–108].

### Cancer-associated fibroblasts

Cancer-associated fibroblasts (CAFs) have a specific definition that includes all fibroblastic, non-vascular, non-neoplastic, non-inflammatory and non-epithelial features in tumorigenesis [109–111]. Usually, the markers of CAFs are α-SMA, fibroblast activation protein-α (FAP-α), fibroblast-specific protein-1 and platelet-derived growth factor receptor-β (PDGFR-β) [112]. In cancer-associated fibroblasts, lncRNA-H19 is highly expressed and promotes stemness and chemoresistance through exosomal transmission. And H19 promotes CRC progression by competitively binding miR-141 and increasing the expression of β-catenin [113]. In recent years, the importance of CAFs in the progression of colitis and CAC has been recognized.

In CAFs, TGF-β signaling is necessary for metastasis by promoting the activation of STAT3 signaling and the secretion of CCL2 and CCL8 during the development of CAC disease [114]. Additionally, Smad7 and Smurf1 (negative regulators in the TGF-β signaling pathway) are decreased in Ikkβ-deficient fibroblasts, which can increase the secretion of hepatocyte growth factor (HGF) and activate CAC progression [115, 116]. In addition, CAFs are associated with ECM remodeling, which can produce ECM constituents and rebuild enzymes, such as TIMPs, MMPs and other proteases [117, 118]. Moreover, another study found that inhibiting the activation of STAT3 in COL1^+^ fibroblasts can reduce tumor growth, while the activation of STAT3 can accelerate the progression of CAC. Thus, reducing the activation of STAT3 in COL1^+^ fibroblasts has the potential to become a therapeutic target for CAC [119].

Additionally, CAFs have been proposed to regulate the tumor microenvironment [120, 121]. CAFs produce various cytokines and chemokines to regulate tumor proliferation, migration and adhesion [122, 123]. In CAC patients, a high level of CCL2 produced by CAFs leads to the formation of macrophages with a tumor-suppressive function [124]. In addition, researchers have found that MCAM is a specific marker for colorectal cancer stromal cells in the CAC mouse model. In MCAM^+^ cancer-associated fibroblasts, MCAM interacts with IL-1 receptor 1 to enhance the NF-κB-IL34/CCL8 signaling pathway and promotes the recruitment of tumor-associated macrophages [125]. The co-expression of CAFs and macrophages has become an important marker of malignant tumors [126, 127].

## Various types of immune cells are correlated with CAC

**Fig. 3:**
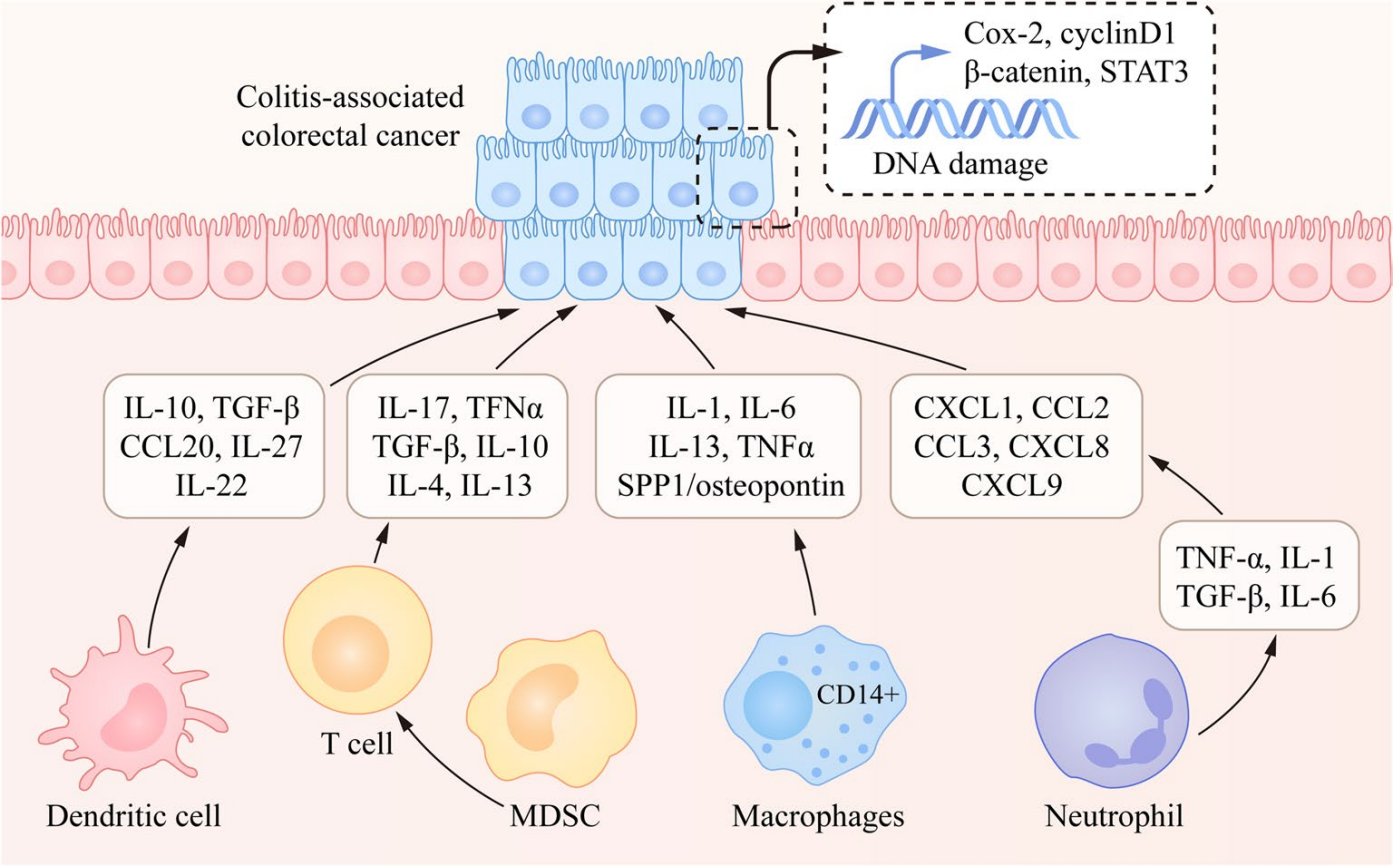
Immune cells regulate the progression of colon cancer by affecting the secretion of cytokines and chemokines. Under inflammatory conditions, immune cells including T cells, dendritic cells, MDSCs, macrophages and neutrophils can secrete cytokines and chemokines to change the tumor microenvironment, thus causing DNA damage and activating tumor-related genes

Several studies have recently shown that colitis-associated colorectal cancer is accompanied by many adaptive immune cells, including T and B lymphocytes, and innate immune cells that contain myeloid-derived suppressor cells (MDSCs), macrophages, dendritic cells (DCs), neutrophils, and NK cells (Fig. 3) [128–131].

T lymphocytes, such as regulatory T cells (Tregs) and helper T cells (Th) are associated with CAC [132, 133]. The removal of CD4^+^ T cells and blocking CCL4 or IL-17 reduced the formation of tumorigenesis caused by myeloid cells [134, 135]. G-CSF/G-CSFR increased the secretion of FoxP3-expressing CD4^+^ and CD8^+^ T cells, while G-CSFR deficiency in T cells increased cytotoxic activity in the tumor microenvironment by producing IL-17A which improved resistance to anti-PD-1 therapy [136–138]. In IBD patients, the blockade of IL-7R reduced colonic inflammation by inhibiting T-cell homing to the gut and altering the activation of effector T cells [139]. Additionally, S1PR4 affected the expression of PIK3AP1 and LTA4H, which is associated with the proliferation and survival of CD8^+^ T cells [140]. In recent years, immunotherapy has played an important role in the treatment of IBD patients [141–143]. FK228 is a histone deacetylase inhibitor that can upregulate the expression of PD-L1 in tumor cells and enhance the antitumor effect by affecting the activity of CD4^+^ and CD8^+^ T cells [144]. In addition, the recruitment of CD4^+^ Th lymphocytes can play an important role in maintaining chronic enteritis in UC and CD patients and promoting colitis-associated colon cancer. Detection in patients with colonic CD showed that high levels of Th1 cytokines lead to an increased risk of CAC in the intestine, whereas the Th2 immune response is directly involved in colitis-associated tumorigenesis by inducing DNA mutations caused by Th2-associated cytokines (i.e., IL-4 and IL-13) in cultured colonic epithelial cells [145, 146]. The expression of FAM64A regulates the differentiation of Th17 but not Th1 cells by modulating the IL-6/STAT3 axis in CAC [147, 148]. Moreover, regulatory T cells are CD4^+^ T cells that express the master transcription factors Foxp3 and CD25, showing immunosuppressive effects by direct cell communication and the release of the cytokines TGF-β and IL-10 [149, 150]. A few components are necessary for the appropriate differentiation and function of Tregs [151, 152]. The analysis of single-cell RNA sequencing data of human colorectal cancer tissues showed that MondoA-thioredoxin-TXNIP axis maintains Tregs and regulates glucose uptake [153]. Erdman et al. suggested that Tregs could develop pathological changes and at the same time reduce tumor formation, which is consistent with a recent study that Tregs have antitumor activity in colitis-associated colon cancer [154].

Myeloid-derived suppressor cells (MDSCs) originate from the bone marrow, which participates in the activation of specific elements and continues multiplication in the pathologic environment [155, 156]. MDSCs are involved in suppressing T-cell immunity via numerous mechanisms, including disturbing T-cell function by reducing necessary nutrients, destroying the normal response of effector T cells, indirectly depressing T-cell function by activating the expansion of regulatory T cells (Treg), and inhibiting the proper expression of L-selectin in naïve T cells [157, 158]. In addition to their immunosuppressive activity, MDSCs play an important role in enhancing angiogenesis [159, 160]. In B16 and RENCA mouse models, STAT3 signaling is considered a fundamental factor in increasing angiogenesis activity in tumor cells. MDSCs and macrophages and the expression of related proteins, such as β-FGF and VEGF, are controlled by STAT3 signaling, which is activated in endothelial cells, facilitates angiogenesis and can be interrupted by STAT3 inhibitors [161]. The pace of colitis development slows down with the treatment of resveratrol in IL-10-deficient mice accompanied by MDSC multiplication and reduced colitis-associated cytokine production [162]. In the development of CAC, MyD88 signaling plays an important role in colonic myeloid cells by regulating the production of pro-inflammatory cytokines and by increasing proliferation and reducing apoptosis in epithelial cells [163, 164]. Recent studies indicate that suppressing the activity of EZH2 promotes MDSC production and ameliorates CAC [165]. Moreover, IL-27 plays an important role in the accumulation of MDSCs and increases tumor cell proliferation, which contributes to CAC development in a murine model [166]. In the AOM/DSS model, there were increased tumor loads and MDSCs in Card9-deficient mice compared with WT mice. In addition, Card9^−/−^ macrophages caused changes in the composition of the intestinal flora, such as a significant increase in *C. tropicalis* [167]. The gut microbiota was associated with the increased CXCL1, CXCL2, CXCL5 expression and MDSC accumulation in tumor tissues [168].

DCs (dendritic cells) play a vital role in the development of colitis because DC deficiency has a strong correlation with the ease of DSS-induced colitis in a mouse model [169, 170]. DCs exhibit a protective effect by activating the rehabilitation of the intestinal epithelium instead of regulating the immune reaction [171]. Another experiment ablated DCs before DSS treatment and inflammation was aggravated, indicating that DCs play a protective role in the initiation of colitis, except for pathogenicity during the progression of tumors [172]. An observation in TGF-β-deficient and IL-10-deficient mice suggested that IL-10 and TGF-β act as determinant factors of DC function in the intestine [173, 174]. In IBD patients, the depletion of DCs in their peripheral blood exacerbates the development of diseases, which regulate the infiltration of MDSCs and cause an increasing number of chemokines, such as CCL20 or MAdCAM-1 (mucosal vascular address in cell adhesion molecule-1) [175–177]. In acute IBD, immature DCs are dramatically decreased, showing that several DC subsets may be absent during disease recurrence. In addition, M-DC8^+^ DCs exist in the subepithelial dome ileum of CD patients, which secrete abundant TNF-α in the treatment of lipopolysaccharide (LPS), contributing to the tumorigenesis of IBD [178]. Moreover, p38α deficiency in DCs influences the activation of Tr1 cells by regulating IL-27 and IL-22 secretion, which plays a pivotal role in the intestinal inflammatory response and tumorigenesis [179]. Additionaly, lymphotoxin signaling induces the expression of IL22BP through the activation of NF-κB in human colorectal tumors and cultured human dendritic cells [180].

Intestinal macrophages are located under the epithelial layer and are considered to originate from classical blood monocytes activated by CCL2/CCR2 [181, 182]. These macrophages express many innate receptors, including the scavenger receptors CD36/CD163, the triggering receptor expressed on myeloid cells (TREM)-2, the C-type lectin receptor CD209 and the FcgR CD64, which promotes the chemotaxis and phagocytosis of bacteria [183, 184]. In a colitis environment, monocytes are moved to the LP and transition to inflammatory macrophages in respond to TLR stimulation and secrete pro-inflammatory cytokines, such as IL-23 [185, 186]. In the human body with Crohn’s disease, CD14^+^ macrophages are most abundant in inflamed tissues, accompanied by the secretion of pro-inflammatory cytokines stimulated by TLR, whereas resident macrophages do not respond to TLR [187–189]. IL-10-deficient macrophages produce higher amounts of prostaglandin E2 after LPS stimulation to impede bacterial killing [190, 191]. Moreover, the deletion of EPRAP in macrophages increased the levels of p105, MEK, and ERK phosphorylation, which led to the activation of stromal macrophages in DSS-induced colitis [192]. CX3CR1-deficient mice showed significantly lower expression of HOMX-1 (an antioxidant and anti-inflammatory enzyme) in adenomatous colon tissue by mediating the CX3CR1 receptor [193]. In primary human and mouse colorectal cancer samples, mTORC2 is only expressed in the adjacent area of macrophages, but not in tumor cells, as mTORC2-deficient macrophages stimulate tumor growth through the cytokine SPP1/osteopontin [194]. BATF2 attenuated inflammation and protected intestinal epithelial cells by inhibiting the transcriptional activation of STAT1/CCL2 and reducing the recruitment of macrophages in colon tissues [195].

Neutrophils accumulate in specific tissues with acute inflammation, acting as the first line of defense, and play an important role in resisting pathogenic microorganisms [196]. In various mouse colitis-associated models, the depletion of circulating neutrophils accelerates inflammation, which suggested that neutrophils are a protective factor in the progression of inflammation [197]. Activated neutrophils produce numerous pro-inflammatory cytokines, including TNF-α, IL-1, TGF-β and IL-6, as well as chemokines, such as CXCL1, CCL2, CCL3, CXCL8 and CXCL9, which result in further recruitment of leukocytes and activation of inflammatory pathways [198]. Additionally, neutrophils increase the production of pro-inflammatory microRNAs (miR-31 and miR-155), which affect genomic instability by modulating replication fork collapse and inhibiting homologous recombination [199, 200]. Moreover, Zhou et al. found that CD177^+^ neutrophils suppress tumorigenesis of epithelial cells and are markedly increased in tumor tissues compared with controls in colorectal cancer [201]. Lin et al. found that the expression of BATF3 was correlated with the poor prognosis of colitis-associated colorectal cancer and promoted the recruitment of neutrophils by modulating the CXCL5/CXCR2 axis [202]. Recently, Zhang et al. identified IRAK-M as an innate suppressor of neutrophils that regulates the activation of STAT1/3/5 and promotes tumor growth [203].

In addition, a few innate immune cells, including basophils and γδ T cells, also play an important role in the pathogenesis of IBD and CAC. The inhibition of AKR1B8 activates innate immunity (including excessive infiltration of basophils and neutrophils) and promotes the occurrence of IBD [204]. γδ T cells, a subset of T cells, are abundant in the intestinal mucosa and maintain epithelial homeostasis [205]. AKR1B8 deficiency leads to increased infiltration of neutrophils and mast cells, as well as a decrease in the number of γδ T cells, thereby disrupting the self-renewal of intestinal epithelium and promoting the progression of inflammation-related colorectal cancer [206].

## Impact of the intestinal microbiome on CAC

**Fig. 4:**
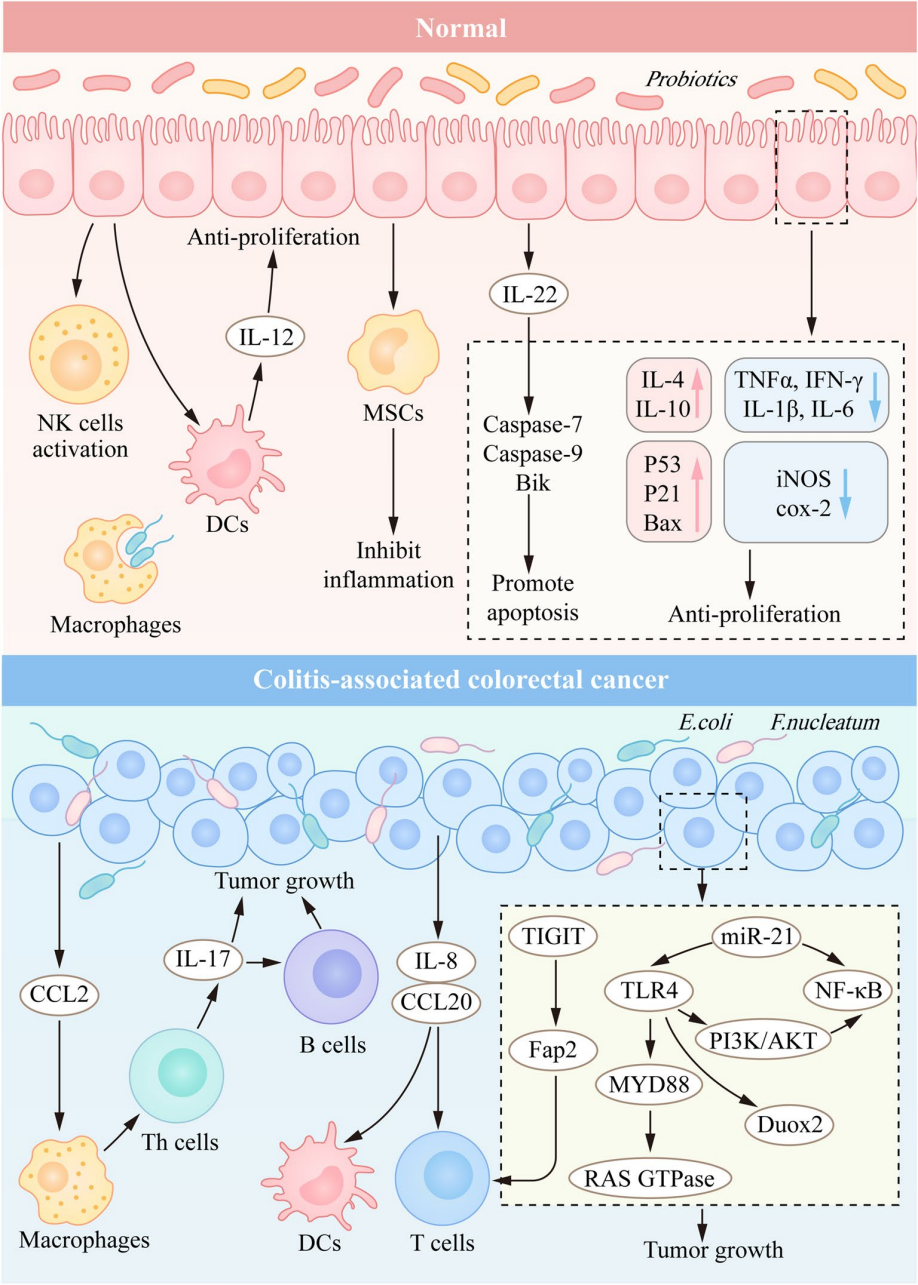
Intestinal flora affects tumor progression. In the presence of probiotics in the intestinal tract, NK cells and macrophages phagocytize the abnormal flora due to the normal state of the body's immune system. At the same time, the secretion of cytokines and chemokines inhibits the abnormal proliferation of intestinal epithelium. In CAC, *E.coli* and other harmful bacteria enter intestinal epithelial cells, regulate various immune cells, and activate tumor-associated transcription factors (such as TLR4, NF-κB and PI3K/AKT), thus aggravating the tumor progression

Gut bacteria play an essential role in regulating gut homeostasis by affecting immunity [207–209]. Dysbiosis of the intestinal microbiome is strongly associated with many intestinal diseases, such as inflammatory bowel diseases (IBD) and colitis-associated colorectal cancer (CAC) [210–212]. With the development of next-generation sequencing technology, an unprecedented view of the intestinal microbiome has been recognized as being involved in intestinal disorders in IBD and CAC patients [213, 214]. Several studies have demonstrated that bacteria promote CAC progression by recruiting macrophages and activating T helper cells [215]. The interaction between the gut and different microorganisms such as pathogenic bacteria, probiotics and fungi will exert or reduce tumorigenic factors in the host [216–218] (Fig. 4).

Harmful microbiota, including *F. nucleatum* and *E. coli* can promote the development of inflammation-related colorectal cancer. *F. nucleatum* was first known as a common anaerobic gram-negative bacterium in the oral cavity and was recently associated with preterm birth, rheumatoid arthritis and colorectal cancer [219]. Recent studies have found that *F. nucleatum* is closely related to the occurrence and development of CAC. Adherence and invasion are important mechanisms in the induction of host defense and host responses. CAC has a strong correlation with the invasiveness of *F. nucleatum* [220, 221]. And *F. nucleatum* was detected in 39.5% of human CAC samples [222]. In addition, *F. nucleatum* increased the expression of tumorigenic genes in CAC by regulating the TLR4-PI3K-AKT-NF-κB pathway [223]. Specifically, *F. nucleatum* increased the expression of miR-21, which represents an increased risk of poor outcomes and may regulate the levels of RAS GTPase by activating the TLR4 signaling pathway and causing the activation of NF-κB signaling in CAC progression [224]. Moreover, *F. nucleatum* can promote the EMT process by activating the expression of the EGFR signaling pathway, thereby accelerating the progression of CAC [221]. Additionally, Rubinstein et al. suggested that *F. nucleatum* promotes the progression of CAC development via its special adhesin FadA and regulation of the E-cadherin/β-catenin signaling pathway [225].

*E. coli* is another commensal bacterium in the human gastrointestinal tract and belongs to the gram-negative and aero-anaerobic bacteria [226]. Various studies have indicated that there is a specific link between *E. coli* and CAC [227, 228]. Adherent invasive *E. coli* (AIEC) is an important pathotype, and the amount of AIEC has increased in colitis-associated colorectal cancer compared with normal tissues [229, 230]. FimH adhesin variants of AIEC can more easily bind to intestinal epithelial cells, which causes the recruitment of dendritic cells (DCs) and macrophages to prevent infection by modulating the secretion of the pro-inflammatory cytokines IL-8 and CCL20 in intestinal epithelial cells [231]. In addition, increased oxygenation of colon epithelium and proliferation of *E. coli* in chemically induced CAC lead to the production of colibactin (an oncogenic factor produced by *E. coli*) [232]. In the CAC mouse model, researchers have found that restricting the proliferation of *E. coli* can alleviate intestinal inflammation and reduce colorectal tumors [27]. Thus, inhibiting the proliferation of *Escherichia coli* is proposed as a preventive strategy for alleviating inflammation-related colorectal cancer.

Probiotics are living microorganisms that have a strong association with diverse health benefits, such as regulating gut microflora, suppressing inflammation and exerting antitumor effects [233–235]. *Lactobacillus* and *Bifidobacterium* are two species of probiotics with tumor-suppressive effects indicated in colorectal cancer cell lines and mouse models [237, 238]. And there are three main mechanisms by which probiotic bacteria prevent colorectal cancer: modulating the immune response, inducing cell apoptosis, and exerting antioxidant activity [239, 240]. The combination of probiotics and prebiotics inhibits the secretion of pro-inflammatory cytokines and inflammation-associated enzymes, such as TNF-α, IL-1β, iNOS and COX-2, and simultaneously upregulates the expression of anti-inflammatory cytokines and pro-apoptotic factors, such as IL-4, IL-10, p53 and p21 [240–242]. The probiotic strain *Lactobacillus casei Shirota* can suppress tumor growth by enhancing the cytotoxicity of natural killer (NK) cells and IL-12 produced by dendritic cells [244, 245]. A recent study revealed that *L. casei BL23* has an immunomodulatory role in CAC by downregulating the cytokine IL-22 and has an anti-proliferative effect by upregulating caspase-7, caspase-9, and Bik [245]. Moreover, MSCs can migrate into the colon and maintain the dynamic balance of intestinal microorganisms by inhibiting chronic inflammation, thereby alleviating CAC progression [246]. Recently, microbiota therapy has been involved in preventing and treating intestinal dysfunction, including IBD, CAC, pathogenic bacterial or viral infection, irritable bowel syndrome (IBS), and antibiotic-associated diarrhea [248, 249]. Oral treatment with zerumbone inhibits the progression of colitis-associated colorectal cancer by reducing the harmful bacteria enterotoxigenic *Bacteroides fragilis* [249].

Although fungi account for only 0.02–0.03% of the intestinal microbiota, the increase in the number of fungi in the intestinal microbiota is an important factor in the development of IBD and IBD-associated colorectal cancer [251, 252]. The fungi in the intestine mainly include *Saccharomyces*, *Candida*, *Penicillium* and *Kluyveromyces*. Compared with healthy individuals, the diversity of fungal species increased in IBD patients [252]. Notably, the abundances of *C. albicans* and *Cryptococcus neoformans* were increased, while *Malassezia sympodialis* and *S. cerevisiae* were decreased in IBD [252]. The use of a single antibiotic, such as cefoperazone, will induce fungal infections (especially *C. albicans*), which affect the composition of bacterial microbiota in the intestine [253]. The immune response against intestinal fungi may affect intestinal inflammation in patients with IBD. Mice with IL-22 deficiency are more likely to be infected by *C. albicans* in the gastrointestinal tract [254]. The secretion of the anti-inflammatory cytokine IL-10 increased significantly under *S.cerevisiae* stimulation, indicating that *S.cerevisiae* plays an anti-inflammatory role. And the amount of *S.cerevisiae* was negatively correlated with a CARD9 SNP allele (rs10781499, ‘A’ allele) in IBD patients [255]. The SYK-CARD9 signaling axis promotes inflammasome activation mediated by commensal gut fungi and thereby inhibits colitis and CAC. In the AOM/DSS-induced mouse model, treatment of mice with antifungal drugs aggravated colitis and CAC [256]. In addition, Malassezia restricta exists on the surface of mammalian skin, which can aggravate colitis in mice and trigger the innate inflammatory response through CARD9 [257]. Moreover, using fungal ITS sequencing, researchers found that some mucosa-related fungi were more abundant in CD patients, and *Malassezia restricta* was especially present in patients with the IBD CARD9 risk allele [258]. These results indicated that targeting specific fungi may become a therapeutic strategy for colitis-related colorectal cancer.

## Therapies for IBD-associated CRC

Currently, surgical resection is the most commonly used treatment for patients with early-stage (stage 0 to II) colorectal cancer. However, chemotherapy drugs such as 5-fluorouracil (5-FU), folinic acid, oxaliplatin and capecitabine are usually used for patients with stage II colorectal cancer. And patients with stage III and IV colorectal cancer are usually treated with chemotherapy and targeted therapy [260, 261]. For IBD-associated CRC, anti-inflammatory therapy in IBD patients may be an effective way to prevent CAC [261].

Chemotherapy has been widely used in colorectal cancer. And 5-fluorouracil is the preferred anticancer drug for the clinical therapy of colorectal cancer. Although 5-FU has therapeutic effects on advanced CRC, the development of drug resistance limits its antitumor effect. And researchers have conducted in-depth research on the molecular mechanism of 5-FU resistance in recent years, hoping to alleviate the development of resistance caused by 5-FU [262]. The inhibition of METTL3 can enhance DNA damage and induce apoptosis in CRC cells by regulating the expression of RAD51AP1, thereby promoting the therapeutic sensitivity of 5-FU. Targeting the METTL3/RAD51AP1 axis has the potential to become a new adjuvant therapy strategy in 5-FU-resistant CRC patients [263]. In addition, the activation of the PI3K/Akt and Wnt/β-catenin signaling pathways can lead to the upregulation of HIF-1α in 5-FU-resistant CRC cells. And the inhibition of HIF-1α combined with 5-FU treatment may enhance the sensitivity of colorectal cancer to 5-FU [264].

The epidermal growth factor receptor (EGFR) gene and its proteins play a key role in promoting CRC tumor growth [265]. EGFR-targeted monoclonal antibodies such as cetuximab and panitumumab have been widely used to treat advanced colorectal cancer [267, 268]. KRAS/NRAS (RAS) wild-type, as well as BRAF/HER2 and MAP2K1 (MEK) mutated CRC patients, are sensitive to anti-EGFR therapy [268]. The expression of EGFR increased in tumor-related myeloid cells and was associated with the outcomes of CRC patients [269]. Anti-angiogenic therapy is also an effective treatment for CRC that targets the vascular endothelial growth factor (VEGF) protein and affects the development of blood vessels during tumor growth. In CAC, inflammation leads to colorectal tumors that are unresponsive to anti-VEGF therapy [138]. To improve the therapeutic effect, multi-target combination therapies are used to treat CAC. The combination of a VEGF inhibitor and a C3b/C4b blocking agent can effectively inhibit angiogenesis and tumor immunity in a mouse CAC model [270].

Immunotherapy has become an effective approach for the treatment of various types of cancers [271]. A growing number of studies indicate that immune checkpoint therapy plays an important role in mediating the immune response mediated by antitumor T cells in the tumor microenvironment [272]. Antibodies targeting PD-1 (programmed cell death 1, also known as PDCD1) and PD-L1 (PDCD1 ligand 1, also known as B7H1 and CD274) are promising strategies in many cancer types, including colorectal cancer. PD-1 is normally seen on the surface of immune cells, such as activated T cells, and is especially overexpressed in inflammatory and tumor conditions [274, 275]. Recently, it has been shown that the loss of PD-L1 in human colorectal cancer cells can lead to chemoresistance [275]. Moreover, CTLA-4 is a co-inhibitory protein usually seen on tumor cells that plays a vital role in immune checkpoint therapy by downregulating the activation and expansion of tumor reactive T cells. Additionally, anti-CTLA-4 promotes antitumor activity by selectively reducing Tregs and simultaneously activating Teffs in tumors [276]. Recent studies have shown that treating mice with therapeutic TNF inhibitors combined with PD-1 and CTLA-4 immunotherapy can improve colitis and antitumor efficacy [278, 279]. In addition, the gene expression of the immune checkpoint molecules Tim-3, LAG-3, Galectin-9, PTPN2 and BTLA is significantly upregulated in patients with colorectal cancer and may become a potential therapeutic target [279–281].

Anti-cytokine therapy, such as anti-TNF and IL-6 therapy, is used in the treatment of IBD, which improves the therapeutic efficiency and prevents the occurrence of CAC (Table 1) [282–284]. Anti-TNFα treatment can block the activation of TNFα receptors and reduce the apoptosis of intestinal epithelial cells, as well as inhibit intestinal permeability. Infliximab, an anti-TNFα drug for IBD treatment, was recently found to facilitate restoration of the colonic barrier of microbiota in Crohn’s disease [285]. As an effective pro-inflammatory cytokine, IL-6 plays an important role in regulating the immune system, such as regulating T-cell activation to control the balance between Th cells and immunosuppressive regulatory T cells in IBD [286]. Also, IL-22 is closely related to mucosal immunity and can directly participate in regulating fungal function. It has been found in humans and mice that IL-22 induces anti-bacterial related reactions, promotes epithelial regeneration, coordinates the endoplasmic reticulum (ER) stress response, and has potential clinical application as a mucosal healing therapy for IBD [40, 288]. In addition, the downstream signaling targets of inflammatory cytokines are also new therapeutic strategies for IBD. Small molecule JAK inhibitors repress the expression of a large variety of pro-inflammatory cytokines, including IL-6, IL-12 and IFN-γ, in the process of IBD [288]. And in the early stage of Crohn’s disease, Smad7 is expressed in a large number of cells in the epithelium and lamina propria of the new terminal ileal mucosa, and the use of Smad7 blocker is helpful to prevent postoperative recurrence [290, 291]. Moreover, a clinical study showed that bone marrow mesenchymal stem cells can be widely used in fistula treatment of patients with Crohn’s disease [291]. And human mesenchymal stem cell-derived exosomes (MSC-Exos) have similar functions as bone marrow mesenchymal stem cells in immune regulation and tissue repair, which can protect against experimental colitis and play a potential role in the treatment of IBD [^293 294^]. T-cell trafficking disruption and transcription factor inhibition are new therapeutic strategies that have recently been carried out in clinical trials. Specifically, targeting β7 integrins and the endothelial adhesion molecule MAdCAM-1 can effectively inhibit the migration of lymphocytes [294]. Recent studies have indicated that α4β7^−^ and α4β7^+^ T cells may upregulate αEβ7 in the intestinal mucosa by activating TGF-β signaling [295]. Another trafficking modulators sphingosine-1-phosphate receptors (S1PRs) is dysregulated on intestinal vascular endothelial cells in patients with IBD, and is involved in the growth, angiogenesis, migration and barrier homeostasis of vascular endothelial cells [296]. In addition, the transcription factor GATA3, whose expression is correlated with the secretion of Th2- and Th9-related cytokines, has been found in UC patients, and the GATA3 DNAzyme may play a role in the treatment of UC patients [297]. Additionally, RORγt (a transcription factor in Th17 cells) can be regulated by a specific strain that induces Th17 cells in IBD patients and promotes the process of colitis [298]. Therefore, new targets of chemotherapy, targeted therapy and immunotherapy have the potential to become effective methods for the treatment and prevention of colitis-associated colorectal cancer.

**Table1:** The clinical experiment of therapies for IBD

Drug	Target	Disease	Phase	No. of patients	Interventions	Study Completion Date
PF-04236921	IL-6	Crohn’s disease	Phase II	250	Drug: PF-04236921 SC injection	February 2015
Esketamine	IL-6	Crohn’s disease	Phase IV	120	Drug: Esketamine; Drug: Placebo	March 2022
UTTR1147A	IL-22	Ulcerative colitis; Crohn’s disease	Phase II	143	Drug: UTTR1147A	July 2022
ABX464	IL-22	Ulcerative colitis	Phase II	32	Drug: ABX464; Drug: Placebo oral capsule	September 2018
Mirikizumab	IL-23	Crohn’s disease	Phase II	191	Drug: Mirikizumab; Drug: Placebo	February 2021
Risankizumab	IL-23	Crohn’s disease	Phase II	121	Drug: Risankizumab IV; Drug: Risankizumab SC; Drug: Placebo	November 2016
Ustekinumab	IL-23, IL-12	Crohn’s disease	Phase II	526	Drug: Placebo; Drug: Ustekinumab	December 2010
Adalimumab	TNF-α	Moderate to severe Crohn’s disease	Phase IV	100	Drug: Adalimumab	January 2017
Golimumab	TNF-α	Ulcerative colitis	Phase IV	112	Drug: Golimumab	September 2019
Certolizumab	TNF-α	Crohn’s disease	Phase IV	20	Drug: Certolizumab Pegol	October 2018
Infliximab	TNF-α	Ulcerative colitis	Phase IV	21	Drug: Infliximab	November 2012
SHR0302	JAK1	Crohn’s disease	Phase II	144	Drug: SHR0302; Drug: Placebos	December 2021
Upadacitinib	JAK1	Crohn’s disease	Phase III	524	Drug: Upadacitinib; Drug: Placebo for Upadacitinib	January 2022
ABT-494	JAK1	Crohn’s disease	Phase II	220	Drug: Placebo; Drug: ABT-494	August 2017
PF-06651600	JAK3	Ulcerative colitis	Phase II	319	Drug: PF-06651600 or Placebo; Drug: PF-06700841 or Placebo; Drug: PF-06700841; Drug: PF-06651600	May 2021
PF-00547659	MAdCAM-1	Crohn’s disease	Phase II	268	Drug: PF-00547659	July 2016
MT-1303	S1P_1_	Crohn’s disease	Phase II	46	Drug: MT-1303	August 2017
Vedolizumab	α4β7	Inflammatory bowel disease	–	24	Drug: Vedolizumab	February 2019
Abrilumab	α4β7	Ulcerative colitis	Phase II	359	Biological: Abrilumab; Drug: Placebo	April 2018

The data was obtained from www.clinicaltrials.gov

## Conclusion

In this study, we reviewed the important functions and crosstalk among different cells, such as IECs, IMCs, immune cells and gut microbiota, in the progression of colitis-associated colorectal cancer. These cells can regulate the occurrence and development of CAC in many ways. Wnt, NF-κB, STAT and other signaling pathways regulate the carcinogenesis of intestinal epithelial cells and stromal cells. Furthermore, various immune cells affect tumor progression by secreting cytokines and chemokines, and the gut microbiota regulates tumorigenesis by influencing the immune response. The interactions among microorganisms, immune cells, and epithelial cells regulate the occurrence and development of tumors. However, the regulatory mechanisms between these cells are still unclear.

In recent years, a few studies have shown that chemotherapy, targeted therapy and immunotherapy have been carried out in mouse models of CAC and have had therapeutic effects. However, clinical research on CAC is rare and requires further in-depth investigation. In addition, many researchers have edited harmful bacteria and used probiotics to alleviate tumor progression and chemoresistance in CAC [300]. However, the target cells and specific molecular mechanisms of these drugs and therapies still need to be further studied. Therefore, in future studies, we should focus on regulating specific signaling pathways in various cell types and clarifying the regulatory mechanisms between different cell types. It is important to investigate the crosstalk between various cells and to design drugs for chemotherapy, targeted therapy and immunotherapy, which provide new approaches for CAC patients.

## Data Availability

Not applicable.

## Abbreviations

CRC: Colorectal cancer
IBD: Inflammatory bowel disease
UC: Ulcerative colitis
CD: Crohn’s disease
IECs: Intestinal epithelial cells
IMCs: Intestinal mesenchymal cells
CAC: Colitis-associated colorectal cancer
ER: Endoplasmic reticulum
JAK/STAT: Janus kinase/signal transducer and activation of the transcription
MSCs: Mesenchymal stem cells
CAFs: Cancer-associated fibroblasts
α-SMA: α-Smooth muscle actin
ECM: Extracellular matrix
EMT: Epithelial-to-mesenchymal transition
FAP-α: Fibroblast activation protein-α
PDGFR-β: Platelet-derived growth factor receptor-β
EREG: Epiregulin
Bmps: Bone morphogenetic proteins
HGF: Hepatocyte growth factor
FGF: Fibroblast growth factors
CTLA-4: Cytotoxic T lymphocyte antigen-4
EGF: Epidermal growth factor
VEGF: Vascular endothelial growth factor
MDSC: Myeloid-derived suppressor cell
DC: Dendritic cell
Treg: Regulatory T cell
Th: Helper T cell
pDC: Plasmacytoid DC
LPS: Lipopolysaccharide
TCS: Triclosan
RA: Rosmarinic acid
AIEC: Adherent invasive E. coli
ETBF: Enterotoxigenic Bacteroides fragilis
NK cell: Natural killer cell
IBS: Irritable bowel syndrome
EGFR: Epidermal growth factor receptor
PD-1: Programmed cell death 1
CCL2: C–C motif chemokine ligand 2
CCL8: C–C motif chemokine ligand 8
